# Resveratrol: Why Is It a Promising Therapy for Chronic Kidney Disease Patients?

**DOI:** 10.1155/2013/963217

**Published:** 2013-12-29

**Authors:** Juliana F. Saldanha, Viviane de O. Leal, Peter Stenvinkel, José Carlos Carraro-Eduardo, Denise Mafra

**Affiliations:** ^1^Graduate Program in Medical Sciences, Federal Fluminense University (UFF), Niteroi, RJ, Brazil; ^2^Clinical Nutrition Department, State University of Rio de Janeiro (UERJ), Rio de Janeiro, RJ, Brazil; ^3^Department of Renal Medicine K56, Huddinge University Hospital, Karolinska Institutet, 141 86 Stockholm, Sweden; ^4^University Hospital Antonio Pedro, Medicine Faculty of Federal Fluminense University (UFF), Niteroi, RJ, Brazil

## Abstract

Resveratrol, a phenolic compound found in various plants, including grapes, berries, and peanuts, shows promise for the treatment of cancer, aging, type 2 diabetes, and cardiovascular diseases. Resveratrol can promote *transcription factor nuclear factor-erythroid 2-related factor 2* (Nrf2) activation, increase the expression level of SIRT-1, which is a sirtuin family protein, and reduce mTOR pathway signaling. This compound has anti-inflammatory properties in that it inhibits or antagonizes the nuclear factor-*κ*B (NF-*κ*B) activity, which is a redox-sensitive transcription factor that coordinates the inflammatory response. Inflammation and oxidative stress, which are common features in patients with chronic kidney disease (CKD), are interrelated and associated with cardiovascular disease and the progression of CKD itself. Because of the modulation of the mechanisms involved in the inflammatory-oxidative stress cycle, resveratrol could play an important role in controlling CKD-related metabolic derangements. Although resveratrol supplementation in theory is a promising therapy in this patient group, there are no studies evaluating its effects. Thus, the present review aims to describe the role of resveratrol in inflammation and oxidative stress modulation and its possible benefits to patients with CKD.

## 1. Introduction

Systemic inflammation and oxidative stress are nontraditional risk factors that are associated with premature cardiovascular disease commonly observed in patients with chronic kidney disease (CKD) [1, 2]. Oxidative stress, which is characterized by an imbalance between oxidative free radical production and antioxidant capacity, appears to be the link between inflammation and cardiovascular disease in dialysis patients [3]. Moreover, in nondialysis CKD stage, oxidative mechanisms can be involved in renal tissue injury, in which the oxidative stress is associated with the progression of CKD itself [4, 5].

Resveratrol, a phenolic compound that is found in various plants, especially including red grapes and their derivatives [6], has demonstrated many beneficial effects, including anti-inflammatory and antioxidant roles by enhancing the production of antioxidant enzymes [7] and modulating nuclear factors involved in the inflammation-oxidative stress cycle [8, 9]. However, no study has investigated the effects of resveratrol on patients with CKD. This review presents exciting evidence of the positive role of resveratrol in inflammation and oxidative stress control, and the study argues how resveratrol may represent an important link in the puzzle of CKD disorders.

## 2. Inflammation and Oxidative Stress in CKD

Inflammation and oxidative stress are interrelated conditions [10]. Oxidative free radicals are generated by phagocytic immune cells in response to inflammatory stimuli and released with proinflammatory cytokines, which in turn amplify the generation of oxidants [11, 12]. To prevent the harmful effects of the oxidative status, enzymatic and nonenzymatic antioxidant systems counteract oxidative free radicals [11].

In CKD, several features might contribute to a persistent state of inflammation and its prooxidant effects, such as proteinuria, reduced cytokine clearance, infections, comorbidities, underlying influences of clinical events, and dialysis-related factors, including membrane bioincompatibility, dialysate backflow, and endotoxemia [13, 14]. Patients with CKD demonstrate antioxidant defense deficiency because of the reduced consumption of vitamins and minerals containing antioxidants, such as vitamin C and selenium [15].

**Figure 1:**
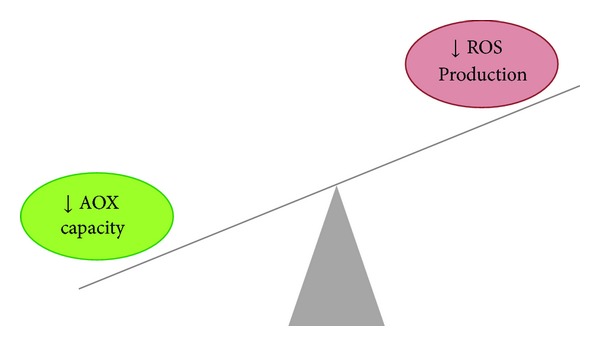
Oxidative stress: imbalance between antioxidant (AOX) capacity and reactive oxygen species (ROS) production.

Oxidative stress results from this imbalance between oxidant generation and antioxidant defense mechanisms (Figure 1), leading to cell and tissue injury [16] and promoting the perpetuation of the inflammation-oxidative stress cycle by activating nuclear factor *κ*B (NF-*κ*B), which is a redox-sensitive transcription factor that mediates the transcription of a large number of inflammatory genes coding for cytokines and adhesion molecules. Thus, when NF-*κ*B is activated, several cytokines are excessively produced, which leads to the formation of oxidative free radicals that establish a vicious cycle between inflammation and oxidative stress [17].

The chronic activation of NF-*κ*B could predispose to atherosclerosis [18] because of its important role in inflammatory phenotypic changes in endothelial and smooth muscle cells [19]. In a recent study, Tilstra et al. [20] showed that NF-*κ*B inhibition delays DNA damage-induced senescence and aging in mice. This transcription factor could be speculated to play a role in the human aging process [20]. Inflammation is a critical mechanism that promotes interlinked fibrosis and cellular injury in the renal interstitium [21], and, additionally, the decrease in renal function is accompanied by increased oxidative stress [22]. In this sense, chronic inflammation and oxidative stress features that are closely associated with NF-*κ*B activation play a key role in the development and progression of CKD and its related disorders [23].

In contrast to NF-*κ*B, *transcription factor nuclear factor-erythroid 2-related factor 2 *(Nrf2) is responsible for the constitutive and inducible expression of antioxidant response element-regulated genes [24] and is recognized to be a major cellular defense mechanism against oxidative stress [17]. When Nrf2 is released from its repressive cytosolic protein Keap 1, after it is translocated to the nucleus, Nrf2 activates genes that encode phase II detoxifying enzymes and antioxidant enzymes, such as glutathione peroxidase and heme oxygenase-1 [17, 25, 26]. In addition to reducing the expression of proinflammatory mediators, including cytokines and adhesion molecules, Nrf2 appears to inhibit NF-*κ*B activation [27] by regulating anti-inflammatory enzymes [17]. Thus, Nrf2 regulates cellular antioxidant responses and inhibits or antagonizes NF-*κ*B actions [17, 24].

Different pharmacological and dietetic compounds are associated with Nrf2 activation. Among food compounds, rutin, quercetin [28], blackcurrant anthocyanins [29], and resveratrol [9] are demonstrated to promote Nrf2 activation.

## 3. Resveratrol

**Figure 2:**
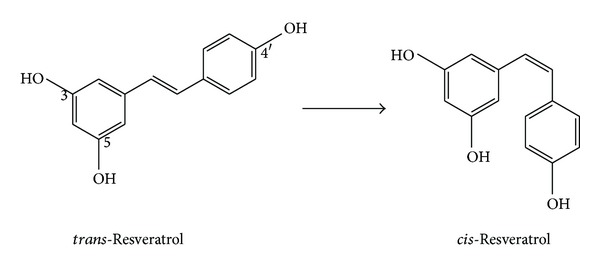
Chemical structure of resveratrol isoforms.

Resveratrol is a metabolite produced in more than 70 plant species in response to environmental stress [6], such as mechanical injury, microbial infection, and UV irradiation. Found in high concentrations in red grapes and their derivatives, resveratrol exists in nature in two isomeric forms (Figure 2): *trans*-resveratrol and *cis*-resveratrol [30]. Two phenol rings are linked by a styrene double bond to generate 3,5,4′-trihydroxystilbene, which was first isolated in 1940 [31]. Although both isomers are biologically active, a majority of biological functions of resveratrol are attributable to *trans*-resveratrol, which is the more stable form. Initially, this compound attracted intense interest in 1992 when several cardioprotective effects were postulated to be associated with red wine [32] which implied that this benefit was an important factor in the French Paradox [33], that is, the observation that the French population has a low incidence of cardiovascular disease, despite having high saturated fat diet [34].

From that time, many biological effects have been assigned to resveratrol; the cardioprotective effects from resveratrol are the most known [35]. Resveratrol seems to improve vascular function by increasing nitric oxide synthesis and inhibiting its degradation [36], in addition to being able to increase the expression of antioxidant enzymes such as superoxide dismutase, catalase, and glutathione peroxidase [7]. Regarding the role of resveratrol in Nrf2 activation, Ghanim et al. [9] evaluated in humans the effects of supplementation with a combination of resveratrol and grape skin polyphenols after a meal rich in carbohydrates and lipids. They observed an increase in Nrf2 and antioxidant enzyme expression, such as glutathione S-transferase [9]. In 2011, Palsamy and Subramanian [8] evaluated the renoprotective nature of resveratrol and observed that resveratrol administered orally to diabetic rats was capable of normalizing Nrf2 renal expression and related antioxidant factors, such as heme oxygenase-1 (HO-1) [8]. In this study, the resveratrol treatment was associated with the normalization of renal function and various inflammatory biomarkers, such as TNF and IL-6, in addition to being related to increased antioxidant activity.

In a randomized clinical trial, Timmers et al. [37] observed that resveratrol supplementation in obese subjects improved glucose tolerance and decreased hepatic steatosis and plasma inflammatory biomarkers [37]. In diabetic patients, resveratrol supplementation contributed to improving insulin sensibility, most likely by reducing oxidative stress [38].

Resveratrol seems to play a role in oxidative stress modulation by reducing NF-*κ*B expression by sirtuins, which are proteins involved in the transcription, apoptosis, and energetic cell regulation [39–43]. The discovery of a homologous sirtuin (SIRT) family of proteins in the mammalian systems led to the realization that these molecules have beneficial effects in metabolism- and aging-related diseases. Sirtuins, NAD^+^-dependent deacetylases, are considered to be central modulators of longevity by playing an antioxidant role in preventing cardiovascular diseases. Until recently, researchers have identified seven homologous genes of the sirtuin family, SIRT-1 to SIRT-7; SIRT-1, with cellular nucleus localization, is the most studied [44, 45]. SIRT-1 expression increases with caloric restriction, during fasting or food deprivation, or when cells are exposed to oxidative stress conditions and DNA damage [46, 47]; SIRT-1 expression seems to decrease under several inflammatory conditions by unknown mechanisms [48]. In humans, Cohen et al. [46] observed that SIRT-1 activation improves the apoptosis resistance of renal embryonic cells [46]; *in vitro* studies suggested that SIRT-1 has an important role in protecting renal medullar cells [44].

In an elegant study, Chen et al. [49] demonstrated that, in rats, resveratrol treatment ameliorated diabetic ketoacidosis and muscle protein degradation by the attenuation of elevated urinary methyl-histidine and plasma branched-chain amino acid levels [49]. In this study, the beneficial effects of resveratrol in diabetic rats were correlated with the activation of hepatic AMP-activated protein kinase and SIRT-1 expression, increases in hepatic and muscular mitochondrial biogenesis, and the inhibition of muscle NF-*κ*B activities. The authors concluded that resveratrol possesses multiple beneficial metabolic effects in insulin-deficient diabetic rats, particularly including effects involved in improving energy metabolism and reducing protein waste [49].

The mechanisms underlying the protective effects of resveratrol on various cardiovascular and metabolic disorders have not been established; however, evidence suggests that the inhibition of the mammalian target of rapamycin (mTOR) signaling pathway could play a role [50, 51]. mTOR is a member of the PI 3-kinase-related protein kinase (PIKK) family that plays a critical role in the regulation of cell homeostasis in response to various upstream stimuli, such as growth factors, nutrients, and stress [52, 53].

**Figure 3:**
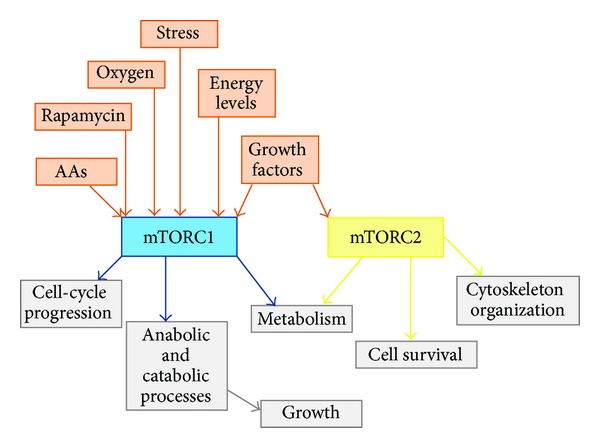
mTORC1 and mTORC2 complexes. AAs: amino acids.

Although several studies have suggested that activation of the SIRT-1 signaling pathway is essential for resveratrol action, Liu et al. [51] demonstrated that resveratrol inhibits insulin- and leucine-stimulated mTOR signaling in a SIRT-1-independent manner [51]. The mTOR kinase nucleates two distinct protein complexes, termed mTORC1 and mTORC2. As presented in Figure 3, mTORC1 is stimulated by stress, oxygen, amino acids, energy, and growth factors that are acutely sensitive to rapamycin. mTORC1 promotes cell growth by inducing and inhibiting anabolic and catabolic processes, respectively, and drives cell-cycle progression and metabolism. mTORC2 is stimulated by growth factors and regulates cell survival, metabolism, and the cytoskeleton [54].

Several findings have indicated that resveratrol can negatively regulate mTOR activity via distinct mechanisms in response to different upstream stimulus [50]. Because mTOR activity is related to inflammatory and oxidative stress processes, its downregulation could attenuate these conditions.

## 4. Resveratrol and CKD

Given that inflammation and oxidative stress are implicated in the pathogenesis of cardiovascular disease in CKD and other complications, compounds capable of attenuating these conditions, such as resveratrol, should attract particular interest in CKD treatment.

**Figure 4:**
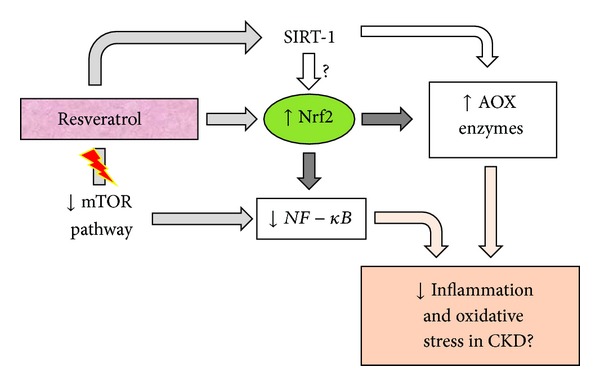
Scheme of resveratrol action mechanism to reduce inflammation and oxidative stress. AOX: antioxidant; CKD: chronic kidney disease.

In mice, Liang et al. (2013) suggested that resveratrol treatment inhibits oxidative stress and renal interstitial fibrosis [55]. Additionally, clinical studies based on polyphenol-containing food supplementation showed improvements in antioxidant activity and lipid profiles in hemodialysis patients [56, 57]. However, until recently, no study has been developed to evaluate resveratrol effects in patients with CKD, although it is plausible that resveratrol could provide several benefits to these patients by reducing inflammation and oxidative stress through SIRT-1 action, mTOR pathway inactivation, and Nrf2 and NF-*κ*B factor modulation (Figure 4).

Although there are no reports of adverse effects related to the use of resveratrol in humans, even at high doses, clinical trials must be developed to explore resveratrol effects in CKD, considering its potential positive effects on systemic inflammation and oxidative stress control. Undeniably, resveratrol supplementation could represent a promising therapy to attenuate the progression of CKD, affect azotemia, and reduce morbidity and mortality by preventing or minimizing the risk of cardiovascular disease in patients with CKD.
